# Supplementary material for: Staphylococcus aureus inhibits the NLRP3 inflammasome in macrophages during the early phases of intracellular infection, but not the late phases
Source: bioRxiv. 2025 Sep 26:2025.09.24.678325. Preprint. [Version 1] doi: 10.1101/2025.09.24.678325 (PMC12485927; doi:10.1101/2025.09.24.678325)
Supplement: Supplement 1 [file media-1.pdf]

***Staphylococcus aureus* inhibits the NLRP3 inflammasome in macrophages during the early phases of intracellular infection, but not the late phases**

Bhagat, et. al.

Supplementary Figure Legends:

Suppl 1: Flow cytometric analysis of *S. aureus*-infected BMDMs. *Aim2+/+;Nlrp3+/+*, *Aim2+/+;Nlrp3-/-, Aim2-/-;Nlrp3+/+,* and *Aim2-/-;Nlrp3-/-* BMDMs were infected with GFP-tagged *S. aureus.* a. Flow cytometry analysis. b. quantitative tabulation of the frequencies of GFP+ cells with respect to total live cells. Data are average values from three biological replicates.

Suppl 2: *S. aureus* suppresses the activation of caspase-1 during the early phases of infection of BMDMs, but to a lesser extent during the late phases - BMDMs were infected with *S. aureus* for up to 48 h. Cells left untreated or treated sequentially with LPS for 3 h and nigericin for 45 min then incubated with 10 µl of 30X FLICA solution followed by the staining with Hoechst 33342. a. FLICA b. Quantitative data. *n* = 3 technical replicates, representative of 3 biological replicates.

Suppl 3: The formation of ASC specks induced by LPS and nigericin depends on NLRP3. S3-A: *Aim2+/+;Nlrp3-/-, Aim2-/-;Nlrp3+/+,* **S3-B:** *Aim2-/-;Nlrp3-/-,* and *Gsdmd-/-* BMDMs were left uninfected or infected with *S. aureus* for up to 72 h. Cells were left untreated or treated sequentially with LPS for 3 h and nigericin for 45 min. ASC specks were visualized under fluorescence microscopy.

Suppl 4: The uptake of Sytox green induced by LPS and nigericin depends on NLRP3. S4-A: *Aim2+/+;Nlrp3-/-, Aim2-/-;Nlrp3+/+,* **S4-B:** *Aim2-/-;Nlrp3-/-,* and *Gsdmd-/-* BMDMs were left uninfected or infected with *S. aureus* for up to 72 h. Cells were left untreated or treated sequentially with LPS for 3 h and nigericin for 45 min. Sytox green was added to the cultures 10 min prior to imaging by confocal microscopy. Data is representative of at least two independent experiments.

Suppl 1

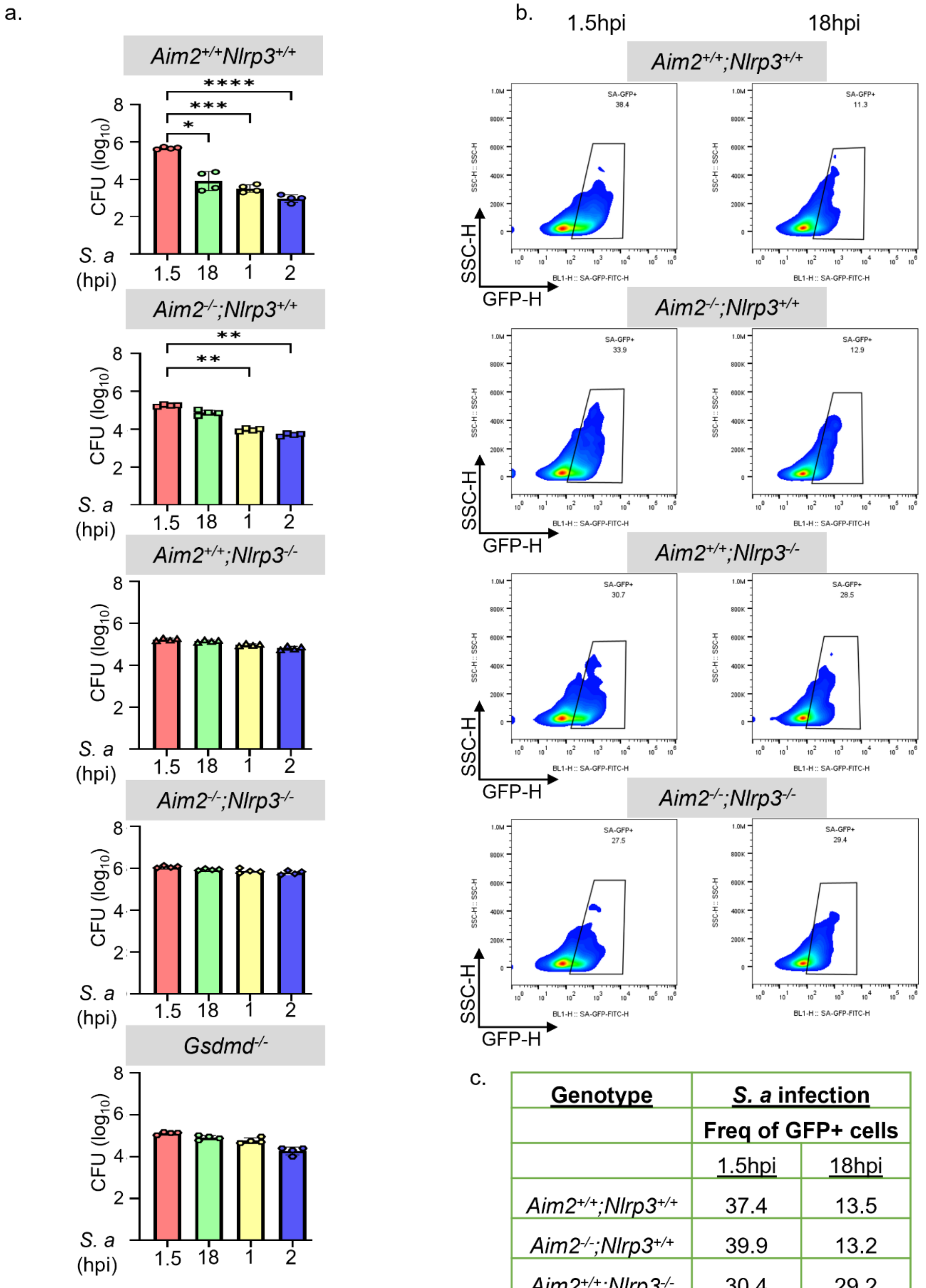

c.

**Genotype**	***S. a* infection**	
	**Freq of GFP+ cells**	
	1.5hpi	18hpi
Aim2+/+;Nlrp3+/+	37.4	13.5
Aim2-/-;Nlrp3+/+	39.9	13.2
Aim2+/+;Nlrp3-/-	30.4	29.2
Aim2-/-;Nlrp3-/-	30.8	28.0

Suppl 2

Aim2$^{+/+}$;Nlrp3$^{+/+}$

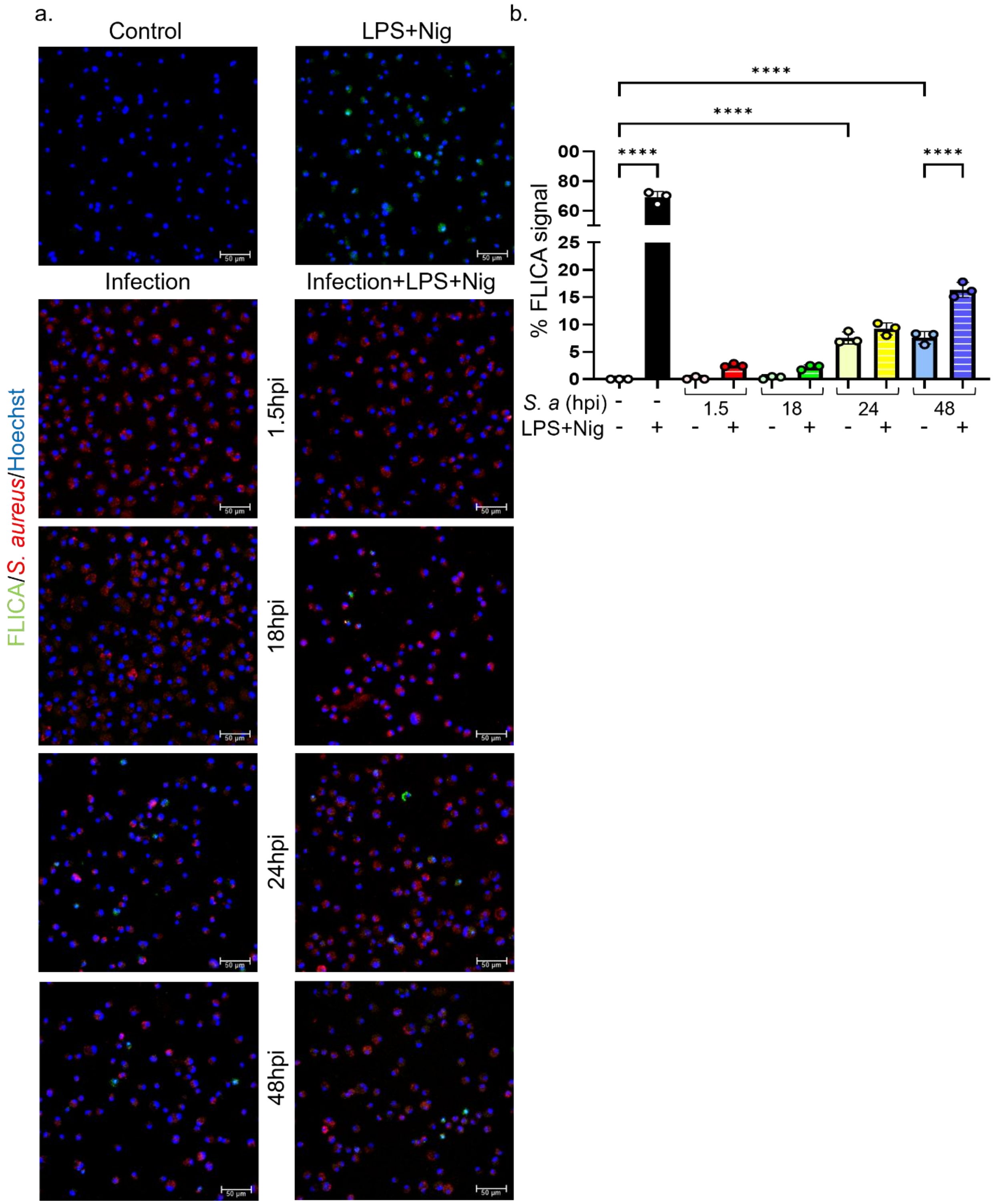

Suppl 3

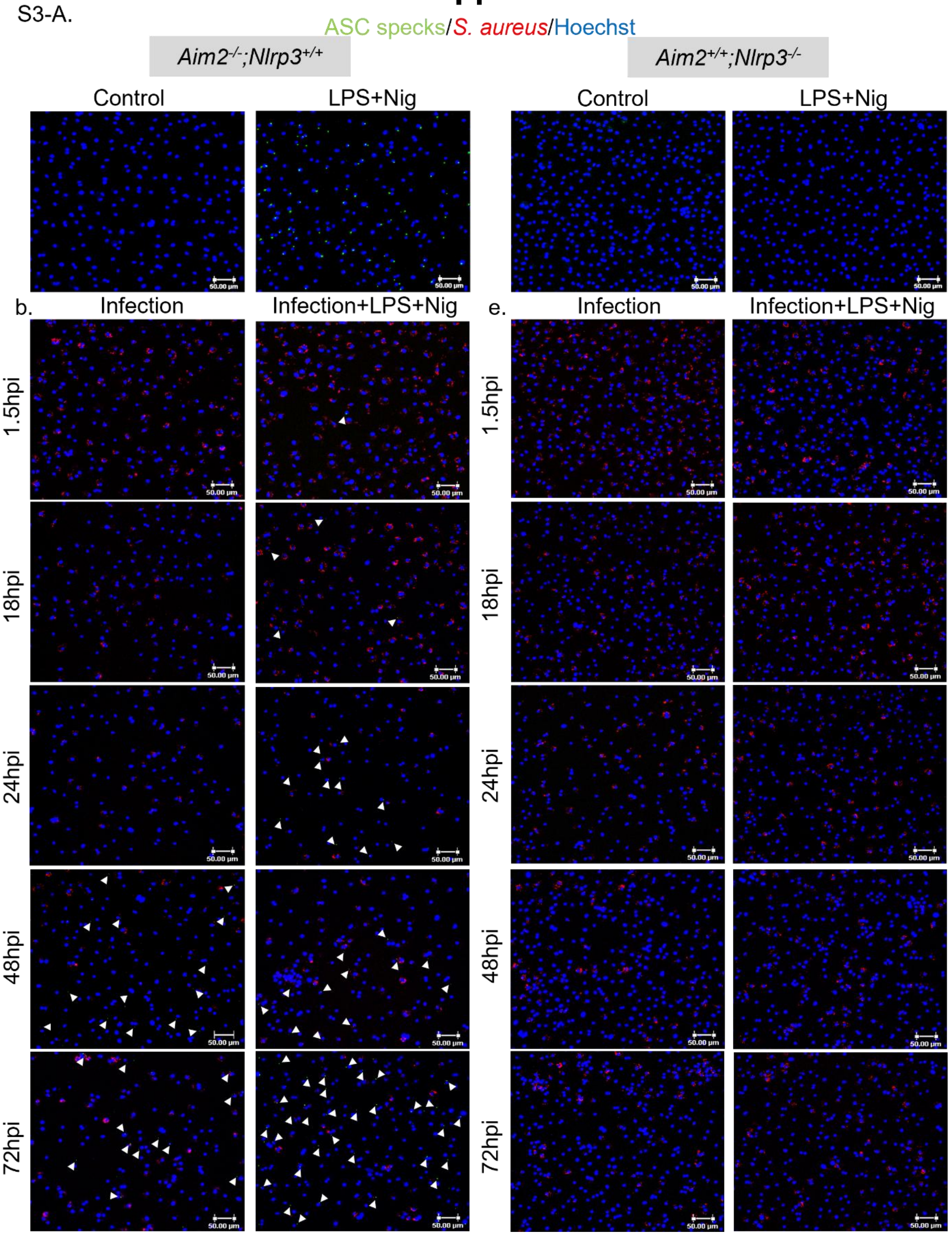
S3-A.
ASC specks/*S. aureus*/Hoechst
Aim2$^{-/-}$;Nlrp3$^{+/+}$
Aim2$^{+/+}$;Nlrp3$^{-/-}$
Control
LPS+Nig
Control
LPS+Nig
b.
Infection
Infection+LPS+Nig
e.
Infection
Infection+LPS+Nig
1.5hpi
18hpi
24hpi
48hpi
72hpi
50.00 µm

Suppl 3

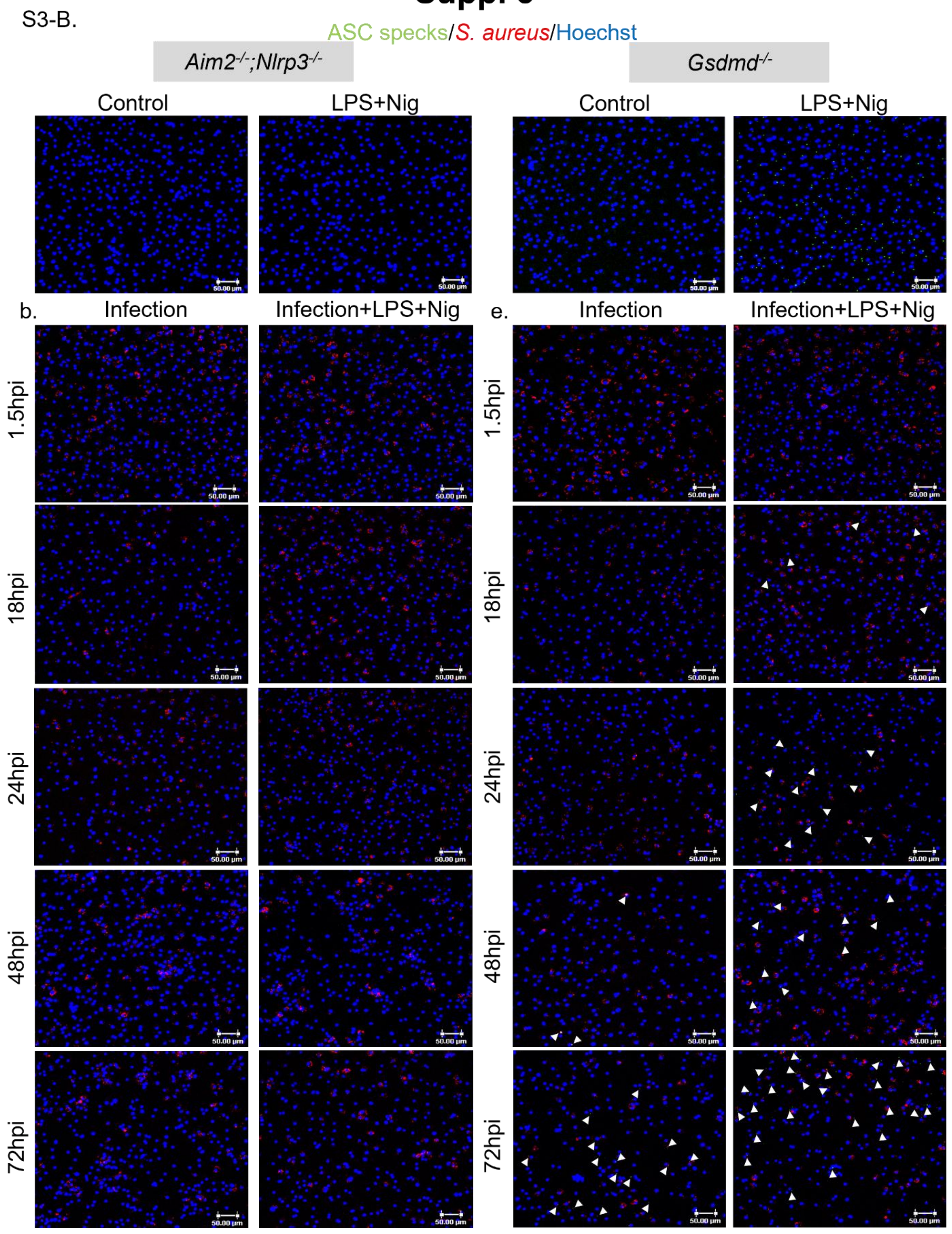
S3-B.
ASC specks/S. aureus/Hoechst
Aim2-/-;Nlrp3-/-
Gsdmd-/-
Control
LPS+Nig
Control
LPS+Nig
b.
Infection
Infection+LPS+Nig
e.
Infection
Infection+LPS+Nig
1.5hpi
18hpi
24hpi
48hpi
72hpi
50.00 μm

Suppl 4

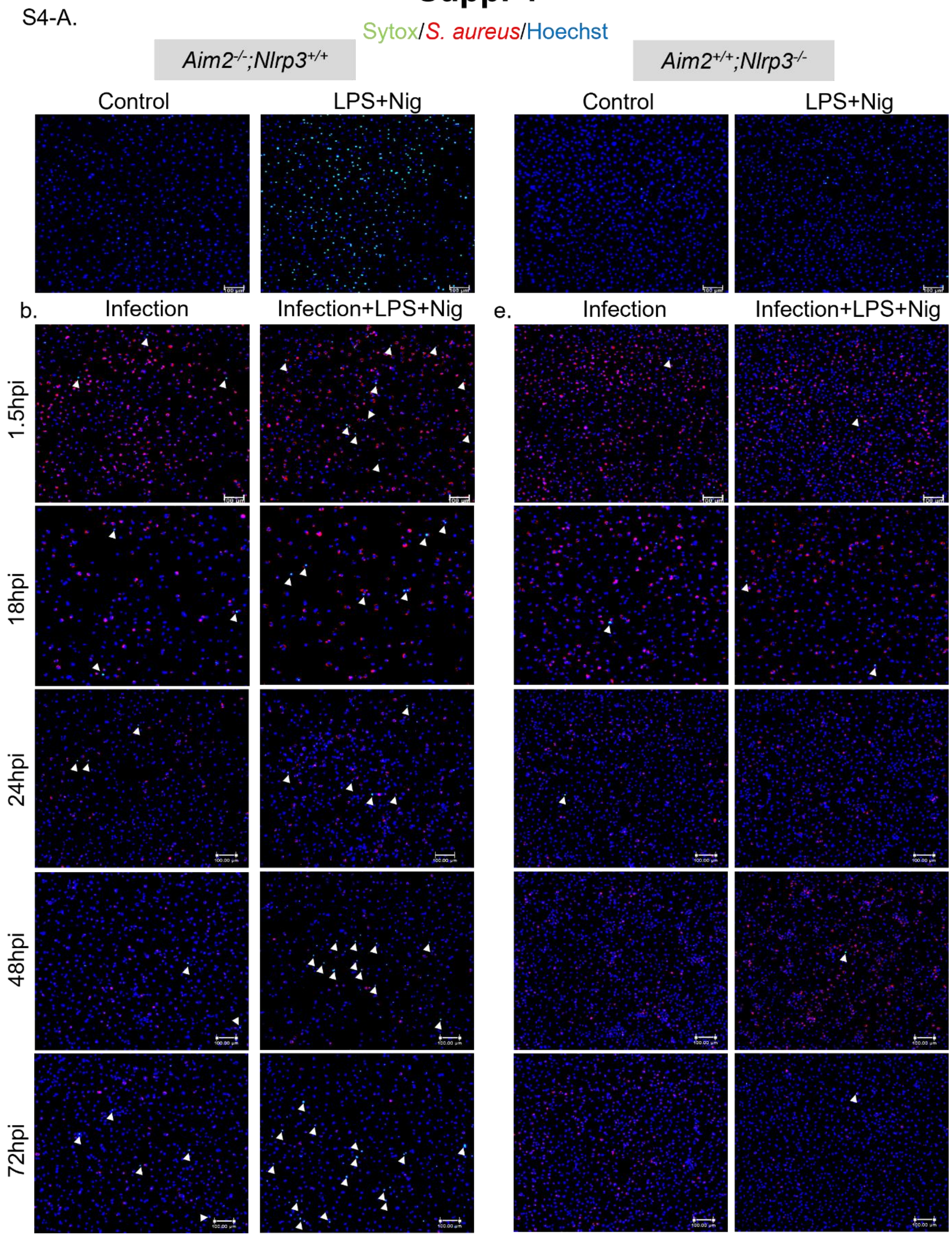
S4-A.
Sytox/S. aureus/Hoechst
Aim2-/-;Nlrp3+/+
Aim2+/+;Nlrp3-/-
Control
LPS+Nig
Control
LPS+Nig
b.
Infection
Infection+LPS+Nig
e.
Infection
Infection+LPS+Nig
1.5hpi
18hpi
24hpi
48hpi
72hpi

Suppl 4

S4-B.

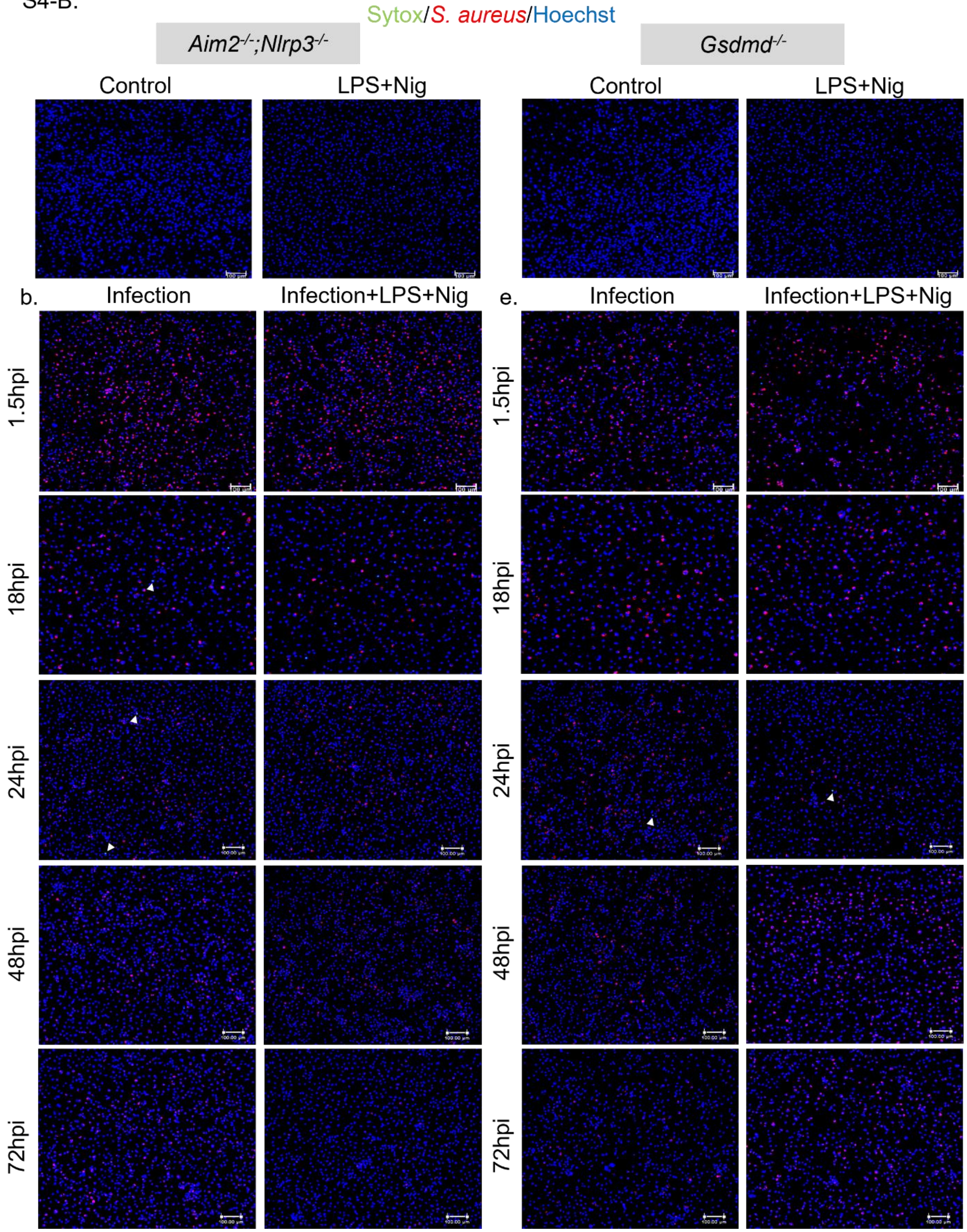

Sytox/*S. aureus*/Hoechst
Aim2$^{-/-}$;*Nlrp3*$^{-/-}$
Gsdmd$^{-/-}$
Control
LPS+Nig
Control
LPS+Nig
b.
Infection
Infection+LPS+Nig
e.
Infection
Infection+LPS+Nig
1.5hpi
18hpi
24hpi
48hpi
72hpi
